# Research and Remedies

**Published:** 1915-07-31

**Authors:** 


### RESEARCH AND REMEDIES.

### The Use of Salvarsanised Serum.

Dr. Marshall Macdonald, of Dunedin, has had some experience of this method of treatment and reports encouraging results. The technique is as follows: A dose of 0.6 gramme of neo-salvarsan in 100 cc. 0.4-per-cent. saline solution is injected directly into a vein, if possible without dissection. An hour later 65 cc. (about 3 ounces) of blood are withdrawn and allowed to coagulate. This is then centrifuged, and the next day 12 cc. of serum are pipetted off and diluted with 18 cc. normal saline solution. This is then kept at a temperature of 56° C. for one hour and then cooled down. Twenty-four hours after the removal of the blood 30 cc. of cerebro-spinal fluid are removed, and then 30 to 35 cc. of the salvarsanised serum and saline are run through a funnel and tube into the spinal canal. The administration may be followed by severe headache or by pains in the limbs, and this may call for the use of morphine. As a rule seven or eight injections as above described are advisable.—*The New Zealand Medical Journal.*

### Radium in Gynæcology.

The most striking effect of radiation in gynæcology is its certain control of excessive uterine hæmorrhage due to metropathies, or to disturbed ovarian function. Cases of women who either bleed continuously or excessively in abnormally prolonged menstrual periods are well known: they are most common in young girls or in women past forty, but they occur at all ages. In some of them a polypoid condition of the endometrium is formed, in others merely a slightly enlarged hard uterus. Even the milder cases suffer from anæmia, and in the more severe forms the patient may die from hæmorrhage unless treated by operation or by radium. Curettage may give temporary relief; a permanent cure is as a rule only obtained by hysterectomy. The use of radium offers a far simpler and safer procedure. Usually a single application of 300 mg. for three hours within the uterus causes a permanent amenorrhœa. A second application should be given in six weeks if the first fails. No inconvenience is caused, and in more than 50 per cent. of the cases the patient is free from hot flushings and other menopausal symptoms. In very young women the treatment is much shorter, and menstruation is rendered normal in type so far as duration and loss of blood are concerned. Rarely has the treatment in such patients to be pushed so as to secure complete amenorrhœa in order to provide complete restoration to health. Hæmorrhage from fibroid tumours of the womb is in similar fashion readily controlled by radium, and, in addition, the tumour will either disappear or be reduced to insignificant proportions. The rate of this shrinking varies much in different patients. After a single radiation one tumour may disappear in two months, another only after six or eight months. Tumours in women past the menopause are readily reduced. The treatment is without danger; a single radiation is usually sufficient. Operation should be limited to fibroids complicated by other pelvic conditions and to those which do not yield to radium treatment. Of these latter some are calcified fibroids, and in others the patient lacks the necessary solvents in her blood. The value of radium in inoperable cancer of the uterus is well known. Further to be noted is the striking benefit produced by two or three applications in pruritus and kraurosis vulvæ.—Dr. Curtis F. Burnam, *Johns Hopkins Hospital Bulletin.*

